# Editorial on the Special Issue on “Mountain Sports Activities: Injuries and Prevention”

**DOI:** 10.3390/ijerph18041405

**Published:** 2021-02-03

**Authors:** Martin Burtscher, Martin Niedermeier, Hannes Gatterer

**Affiliations:** 1 Department of Sport Science, University of Innsbruck, 6020 Innsbruck, Austria; martin.niedermeier@uibk.ac.at; 2 Institute of Mountain Emergency Medicine, EURAC Research, 39100 Bolzano, Italy; hannes.gatterer@eurac.edu

## 1. Introduction

Recreational outdoor activities like mountain sports are gaining large popularity all over the world and particularly in the Alpine regions. Such activities include sports like downhill or cross-country skiing, ski mountaineering, mountain hiking, rock or ice climbing, high-altitude mountaineering, mountain biking, paragliding, and many more. Although considerable health benefits associated with physical activity, especially when performed on a regular basis, are widely accepted, certain mountain sports also bear an inherent risk of injury due to both objective and subjective risk factors. Thus, knowledge on those risk factors forms an important foundation for the development of appropriate preventive measures in order to reduce accidents and emergencies, and to increase the health benefits of mountain sports.

Therefore, this Special Issue on “Mountain Sports Activities: Injuries and Prevention” was aimed to seek research papers on injuries occurring during various types of recreational mountain sport activities, focusing on risk factors, underlying causes and mechanisms, and outcomes of and/or suggestions for prevention studies. When we made this call for submissions, we did not expect such an active interest in this topic. Finally, 22 articles have been published, covering a broad spectrum of information on the risks of illness, injury, and even mortality associated with mountain sports activities. It is a pleasure to summarize the main messages provided by these papers, but there is also a need to identify the strengths and weaknesses of the methodological approaches applied and to derive ideas for future studies potentially contributing to safety of, and health benefits from, being physically active in mountainous regions.

## 2. Death Risk in Mountain Sports Activities

Sport-related deaths are always unexpected catastrophic events, which have a devasting impact on families, friends, and sporting colleagues. Thus, the prevention of such events is of utmost importance. Four studies (two reviews and two original papers) focused on available data on the death risk when performing mountain sports primarily practiced during the winter [1,2] or the summer season [3,4]. With the exception of ski touring the death risk turned out to be low across different winter sports (0.3–0.8 deaths per 1 million exposure days). In contrast, death rates for mountain sports in the summer season varied dramatically, being much higher in high-altitude climbing, mountain, rock and ice climbing, and paragliding, when compared to mountain hiking, trekking, or mountain biking (below 1 death per 10,000 persons at risk). Mortality rates were also relatively low for climbers using via ferratas, where the death risk was highest, but preventable, for those climbing unsecured easy-to-climb sections [4]. Traumatic events like falls were predominant causes in climbers and downhill skiers, avalanche burial and/or high-altitude illness in ski tourers and in high-altitude climbers, and non-traumatic events like sudden cardiac death in sports with high participation of older individuals (downhill and cross-country skiing, and hiking). Whereas insufficient mountaineering skills and/or preparation, and overestimation of their own capabilities were considered as major reasons for traumatic deaths, avalanche burial, and high-altitude illness, lack of physical fitness and pre-existing diseases seem to be associated with non-traumatic (cardiovascular) deaths [1,3]. Unfortunately, information on the number of persons at risk, on environmental conditions, and on individual characteristics associated with the accident/emergency are often lacking, but would be important for proper risk calculations. Although every effort has been made by alpine clubs, institutions dealing with skiing and mountaineering safety, and sports physicians, more appropriate studies are needed to evaluate the effects of preventive strategies.

## 3. Frostbites, Skeletal Muscle, and Kidney Injuries

The hostile environmental conditions at high altitude or when performing mountain sports in the winter are associated with the risk to suffer from frostbite and/or hypothermia, frequently resulting in adverse long-term consequences or even death. Carceller and colleagues evaluated factors related to the risk of amputation in patients who suffered from severe frostbite [5]. Regression analysis revealed the mountain range, years of expertise in winter mountaineering, time elapsed before rewarming, and especially altitude as the most important factors for a poor prognosis.

Ultra-endurance events in the mountains are gaining increasing popularity, but (mostly reversible) skeletal muscle and kidney injuries are common observations in participants. However, pathophysiological mechanisms explaining individual causes of those injuries are not well explored. In this regard, Rojas-Valverde and colleagues evaluated the importance of physical external workload, placing inertial measurement units at different body sites of ultra-endurance runners [6]. The authors demonstrated that the impact of external workload on muscle and kidney injury markers was associated with body sites, i.e., lumbar region L1–L3 sites with kidney injury, supporting the idea of mechanical kidney injury during trail running.

## 4. Falls in Mountain Hiking

Fortunately, most accidents do not lead to death. Different studies in this special issue deal with injuries or accidents occurring during the various mountain sport activities. Two studies specifically focused on falls during mountain hiking [7,8], and one analyzed risk perception during this activity [9]. The study of Faulhaber et al. (2020) was a 3-year (2016–2018) prospective trial that investigated non-fatal accidents caused by falls during hiking in Tyrol (Austria). Data were gathered from questionnaires sent to the injured hikers by the Austrian Alpine Police, who routinely document all mountain accidents with an emergency or local mountain rescue call. The study revealed that among hikers sustaining a fall during mountain hiking a high percentage affected people older than 50 years (approximately 70%). Additionally, a high percentage of the victims suffered from visual impairment. Interestingly, the type of shoes does not seem to have any influence. In line with their data, the authors recommend regular eye examination and, if necessary, the adjustment of visual aids [7]. The most relevant limitations reported by the authors were the response rate of only about 50%, which may have resulted in a selection bias, and missing registered cases (e.g., self-evacuation). The second study focusing on falls was performed on Mount Fuji (3776 m), and thus characterizes falls in a specific area and on a specific terrain. It needs to be remembered that the terrain at Mount Fuji is characterized by long gravel, sand, and volcanic ash. On different occasions, Uno et al. (2019) distributed questionnaires to Japanese hikers coming back from the summit with the focus to evaluate frequency of falls and potential risk factors. The authors report that 30% of hikers (n = 167) experienced a fall with 50 cases of injuries (e.g., knee pain, sprained ankle, and scratch). The main cause for falls were slips. Female sex, no prior climbing experience on Mount Fuji, and not staying at a lodge are the main factors related to an increase in the risk of falls [8]. As evidenced by the authors, the study outcomes are limited to a specific area (i.e., Mount Fuji) and population (Japanese with mostly little climbing experience). A further study included in the special issue did not directly investigate hiking accidents or injuries, but the risk perception of hiking. The risk perception of hiking, besides others, is thought to determine the readiness to practice mountain hiking [9], and hence has an impact on the number of people at risk. Similar to the study of Uno et al. (2019), the result of the survey is limited to the assessed area and population (i.e., Chinese living in city of Guilin). In this investigation, questionnaires consisting of three parts, (i) sensation seeking of the hikers, (ii) perceived risk of hiking, and iii) demographic characteristics, were handed out to hikers who responded to a call posted on social networks and additional to college students (the results of the latter population will not be addressed further here). 174 questionnaires of hikers were included in the final analyses. The study identified two dimensions of perceived risk towards hiking, i.e., physical risk and psychological risk. Physical risk reflects concerns about safety, and psychological risk reflects concerns about negative psychological experience. Data show that the higher the perceived physical risk is, the more the individual is inclined to an easy hiking route (i.e., less than 20 km, not very steep, and without difficulties). Surprisingly, hikers who perceived higher psychological risk were more likely to choose a difficult route. This finding could reflect the concern of hikers that the experience would not meet their high expectation. The limitations of the study were addressed by the authors and include the limited sample size, missing information on the external validity of the questionnaire, and the restriction of the outcomes to a limited area [9].

## 5. Mountaineering and Climbing

Moving in steeper and rockier terrain is called mountaineering and can culminate in climbing activities. Of course, the latter can also be performed indoors. One study in this special issue analyzed climbing accidents leading to multisystem trauma recorded in the International Alpine Trauma Registry (IATR), and furthermore included a systematic literature review concerning this topic [10]. A second study examined youth climbers’ awareness of the most common youth climbing injuries [11]. Rauch et al. (2019) outlined that a considerable amount of studies investigated climbing-related overuse injuries or acute injuries involving a single body part, e.g., the fingers or shoulder, while data on multisystem trauma is scarce. The IATR, a transnational platform for the prospective collection of climbing accidents encountered in mountainous or remote areas, included 37 multisystem trauma cases that occurred in parts of Italy, Austria, and Switzerland from 2010 to 2019 (one year missing). Fall onto the ground was the most common mechanism leading to trauma (51.4%), followed by fall in a snowfield (10.8%), fall in a crevasse (8.1%), and hit by a stone (5.4%). Head/neck, chest, and abdomen have been the predominant sites of injury. Interestingly, the mean body temperature of the patients was approximately 35 °C. As hypothermia is an independent risk factor for mortality in trauma patients, the authors suggest that the prevention of hypothermia is key and can also be achieved by companions while awaiting professional rescue [10]. The main limitation mentioned by the authors is incomplete data in the register. For information on the systematic literature review included in this publication, the interested reader is referred to [10]. Meyers et al. (2020) identified a paucity of literature examining youth athlete’s awareness about youth climbing injuries and therefore sought to fill this gap. The authors distributed questionnaires to elite youth climbers aged 8 to 18 years who participated in the 2017 USA Climbing Youth Sport and Speed National Championships. Survey questions consisted of the following four types of questions: (1) Demographic, training, and injury questions; (2) injury ranking questions; (3) injury (factual) knowledge; and (4) safe training practices. 267 of the 613 athletes completed the survey, and a widespread misperception about finger growth plate injuries and A2 pulley injuries was recorded. The youth climbers believed the most common climbing-specific injury among skeletally immature climbers is the A2 pulley injury, however, according to the literature, this should be growth plate injuries to the finger. A further finding of the study was that the majority of the climbers do not know the safe age to start double dyno campus board training. The authors concluded that educating young people, coaches, and parents about finger injuries would raise awareness of the risks and potentially lead to the introduction of safe training regimes and rules that could help reduce the incidence of growth plate injuries [11]. The authors acknowledge that all results are derived from self-reported data. The youngest may not have been familiar with the medical terms and may have asked the parents, which may have introduced error by including parents’ knowledge in the analysis. Furthermore, the questionnaire still lacks validation. It is important to mention that the response rate was approximately 44%. Two studies published in the present Special Issue did not directly focus on injuries, but on flexibility and masticatory muscle activation; however, flexibility training is an important component in injury prevention programs and higher masticatory muscle activation was hypothesized by the authors as a proxy for being able to reduce allostatic load more efficiently. Ginszt and colleagues reported higher masticatory muscle activation during maximal voluntary clenching in sport climbers compared to non-regularly active persons [12]. As this difference was not found for resting masticatory muscle activation, the authors hypothesized that teeth clenching might be conducted regularly during climbing. Draga et al. [13] aimed to investigate the relationship between climbing performance and several flexibility tests in climbers of advanced to higher elite level. The authors reported moderate-to-large correlation coefficients between climbing performance and hip flexibility [13]. Higher climbing performance was associated with larger hip abduction flexibility assessed by standard, non-climbing specific flexibility tests. Interestingly, this association was not found in climbing specific flexibility tests.

## 6. Injuries in Backcountry Skiing

Backcountry skiing, also called ski mountaineering, is another alpine sport that has gained popularity in recent years. With the increasing number of participants equipment also became better, which prompted Gasser (2020) to explore whether those improvements reduced the severity of injuries in Switzerland. The author analyzed all mountain emergency cases of the Swiss Alpine Club central register for the period 2009 to 2018 (i.e., 3044 cases (953 female and 2091 male)). The term “mountain emergency” covers all events where mountaineers claim the help of mountain rescue services and were classified as falls, blocking, losing way, avalanches, illnesses, and other. In the 10-year observation period, the estimated average annual increase in all emergencies was 3.5% per year, which was almost the same as the increase in the members in the Swiss Alpine Club during the respective time (around 4%). Moreover, data suggest a small but significant increase in the severity of mountain emergencies over the 10-year period over all events. The increase in severity despite better equipment and possibilities of tour planning (online access to information on avalanche and snow conditions) was explained by a potential false sense of security of less-experienced backcountry skiers [14]. Similar to the studies analyzing mortality risk, the total number of people at risk is difficult to assess, and the estimate according to the number of members belonging to different Alpine Clubs may be subject to some error.

## 7. Jumping and Drowning in Canyoning Activities 

Canyoning, compared to other mountain sports, may be considered a niche sport in the mountains, even though it is becoming increasingly popular. The sport requires diverse skills, such as hiking and climbing up and down wet and dry rocks, roping techniques like rappelling, as well as swimming, sliding, and jumping off cliffs or waterfalls. Principal risks encompass falls onto rocks, drowning, and hypothermia, yet comprehensive literature on dangers and injury patterns in canyoning is scarce. This prompted Ströhle et al. (2019) to analyze nationwide (i.e., Austria) canyoning data from 2005 to 2018. Data for analysis were gathered from the national registry for mountain accidents of Austria, which includes anonymized data on accidents and rescue operations in the Austrian Alps, as well as from the information system at Innsbruck Medical University Hospital. In the analyzed time period, 297 canyoning accidents involving 471 people were recorded in the national registry and 58 in the hospitals information system (9 of them requiring professional rescue). Data revealed that jumping, rappelling, sliding, and stumbling were the most common causes of canyoning accidents, and thus require increased caution. Injuries most commonly involved the lower extremity, followed by the shoulder and the spine. Death (n = 9) was mainly caused by drowning. Nearly half of the rescue operations required a helicopter to be dispatched and a quarter of all canyoneers were uninjured, but needed rescuing [15]. The main limitations outlined by the authors concern completeness of the data and missing cases due to self-rescuing (minor injuries) and again the lack of knowledge about the numbers of people at risk.

## 8. Potential Risk Factors for Injuries in Skiing and Snowboarding

Seven studies of the Special Issue were conducted on risk factors for injuries in skiers and snowboarders. Six studies focused on internal risk factors including, but not limited to, knowledge, behavior, or anthropometric characteristics of ski and snowboard practitioners. Using a cross-sectional design, Carus and Castillo interviewed both snowboarders on ski slopes and freestyle skiers/snowboarders in snow parks on their knowledge of safety rules according to the International Ski Federation (FIS) [16,17]. Their results revealed a lack of safety knowledge, especially in younger and less experienced practitioners. Since 63% of freestyle skiers/snowboarders [17] and 38% of the slope snowboarders [16] were unaware of the existence of FIS rules, campaigns to more effectively communicate the safety rules in ski resorts are recommended by the authors. In another study, snowboarders showed a higher sensation seeking and a self-reported alcohol consumption compared to downhill skiers or ski tourers [18], which strengthens the assumption that snowboarders remain an important risk group for injury prevention programs. However, sensation seeking seems to be an important behavioral variable generally, since higher sensation seeking scores were found in injured winter sports practitioners compared to uninjured winter sports practitioners [18]. Helmet usage is strongly recommended in nearly all mountain sports activities. However, helmet usage might have the potential to result in a riskier behavior based on the perception of a higher level of security due to the helmet. This assumption was tested in the study of Ruedl et al. [19], who did not find evidence for a positive association between helmet usage and anterior cruciate ligament injury risk in skiers. Instead, lower skill level, riskier behavior, and older age were found to be associated with higher injury risk. The limitations of questionnaire-based studies (e.g., recall bias, untruthfully answered questions) have to be taken into account in these studies. While the previous studies focused on recreational winter sport participants, a one-season prospective study was conducted in elite youth ski racers [20]. The authors identified later maturation and larger changes in anthropometric characteristics as potential risk factors for injuries over the season. Conversely, improvement in jump coordination performance over the season was associated with a lower risk for injury [20].

In a laboratory study, Promsri et al. [21] investigated leg dominance as a possible injury risk factor based on previous reports on a higher injury risk of the non-dominant leg compared to the dominant leg in female skiers. Their findings include significant differences between legs in movement coordination and control, but not in leg strength. This provides support for a leg discrepancy in sensorimotor control as a more plausible injury mechanism compared to a difference in leg strength between the dominant and non-dominant leg [21]. Another laboratory study focused on potential external risk factors for injuries [22]. Specifically, effects of visual (reduced sight similar to snowfall) and auditory perturbations (listening to music) on ski-specific balance were tested. Although the findings suggest a different coping with the environmental perturbations between sexes, no significant main effects of visual and/or auditory perturbations on balance were detected [22]. Therefore, future laboratory studies in this field might consider a less simplistic setting to mirror the setting of a real skiing condition more realistically.

The studies in the present Special Issue focused predominantly on the risks associated with mountain sports activities, which need to be accepted as an inherent part of the mountainous environment, where the activities are conducted. These risks might be part of the fascination and at least one reason for the increasing popularity of the mountain sports activities. However, in the synthesis, the risks should not displace the associated health benefits of mountain sports activities. Nevertheless, it is important to make all efforts to make activities in mountainous regions as safe as possible. With the support of all contributing authors, we hope to have made a small contribution by the information provided in this Special Issue.
